# Preventive zinc supplementation in developing countries: impact on mortality and morbidity due to diarrhea, pneumonia and malaria

**DOI:** 10.1186/1471-2458-11-S3-S23

**Published:** 2011-04-13

**Authors:** Mohammad Yawar Yakoob, Evropi Theodoratou, Afshan Jabeen, Aamer Imdad, Thomas P Eisele, Joy Ferguson, Arnoupe Jhass, Igor Rudan, Harry Campbell, Robert E Black, Zulfiqar A Bhutta

**Affiliations:** 1 Division of Women & Child Health, The Aga Khan University, Karachi, Pakistan; 2 Centre for Population Health Sciences, University of Edinburgh, UK; 3 Department of International Health and Development, Tulane University School of Public Health and Tropical Medicine, USA; 4 Department of International Health, Bloomberg School of Public Health, Johns Hopkins University, Baltimore, USA

## Abstract

**Background:**

Zinc deficiency is commonly prevalent in children in developing countries and plays a role in decreased immunity and increased risk of infection. Preventive zinc supplementation in healthy children can reduce mortality due to common causes like diarrhea, pneumonia and malaria. The main objective was to determine all-cause mortality and cause-specific mortality and morbidity in children under five in developing countries for preventive zinc supplementation.

**Data sources/ review methods:**

A literature search was carried out on PubMed, the Cochrane Library and the WHO regional databases to identify RCTs on zinc supplementation for greater than 3 months in children less than 5 years of age in developing countries and its effect on mortality was analyzed.

**Results:**

The effect of preventive zinc supplementation on mortality was given in eight trials, while cause specific mortality data was given in five of these eight trials. Zinc supplementation alone was associated with a statistically insignificant 9% (RR = 0.91; 95% CI: 0.82, 1.01) reduction in all cause mortality in the intervention group as compared to controls using a random effect model. The impact on diarrhea-specific mortality of zinc alone was a non-significant 18% reduction (RR = 0.82; 95% CI: 0.64, 1.05) and 15% for pneumonia-specific mortality (RR = 0.85; 95% CI: 0.65, 1.11). The incidence of diarrhea showed a 13% reduction with preventive zinc supplementation (RR = 0.87; 95% CI: 0.81, 0.94) and a 19% reduction in pneumonia morbidity (RR = 0.81; 95% CI: 0.73, 0.90). Keeping in mind the direction of effect of zinc supplementation in reducing diarrhea and pneumonia related morbidity and mortality; we considered all the outcomes for selection of effectiveness estimate for inclusion in the LiST model. After application of the CHERG rules with consideration to quality of evidence and rule # 6, we used the most conservative estimates as a surrogate for mortality. We, therefore, conclude that zinc supplementation in children is associated with a reduction in diarrhea mortality of 13% and pneumonia mortality of 15% for inclusion in the LiST tool. Preventive zinc supplementation had no effect on malaria specific mortality (RR = 0.90; 95% CI: 0.77, 1.06) or incidence of malaria (RR=0.92; 95 % CI 0.82-1.04)

**Conclusion:**

Zinc supplementation results in reductions in diarrhea and pneumonia mortality.

## Introduction

About 30% of the world’s population is zinc deficient [1], most prevalent in children under 5 years of age in developing countries [2]. Zinc deficiency is associated with impaired immune function which results in an increase in morbidity due to infections, growth retardation, hypogonadism and cognitive dysfunction [3,4]. Zinc deficiency is primarily related to the consumed diet; zinc is most abundant and easily absorbable from animal proteins, whereas consumption of vegetable and cereals decreases its absorption due to binding of zinc to phytates [5,6].

Over the past two decades, strong evidence has come forward from multiple randomized controlled trials, in both developed and developing countries, showing an effect of zinc in decreasing morbidity and mortality in children due to gastrointestinal and respiratory infections [7,8]. More recent trials from sub-Saharan countries have also shown an effect on malarial morbidity [9]. This effect of zinc against infectious diseases is therapeutic as well as preventive. Previous reviews by the Zinc Investigators’ Collaborative Group in 1999 [10] and by Aggarwal et al. [11] have studied the effect of preventive zinc supplementation on diarrheal and respiratory morbidity. The current systematic review presents the effect of zinc supplementation on mortality in children less than 5 years of age in developing countries. The evidence for the effect of zinc supplementation on cause-specific mortality and morbidity is assessed for diarrhea, pneumonia and malaria according to guidelines set by the Child Health Epidemiology reference Group (CHERG) for input into the Lives Saved Tool (LiST) model [12].

## Methods

### Searching

A comprehensive literature search was conducted using the following search strategy: zinc AND (infection OR diarrhea OR pneumonia OR ARI OR malaria OR morbidity OR mortality OR death) in electronic bibliographic databases i.e. PubMed, the Cochrane Library and WHO regional search engines, including articles cited up to September 25^th^ 2010 . The limits used were “Humans” and “Randomized controlled trials”. Additional studies were obtained through hand search of references from identified studies. One author reviewed the titles and abstracts to identify controlled studies conducted in developing countries in which supplemental zinc was administered and outcomes on mortality or morbidity were reported. Two authors independently assessed eligibility using the pre-defined inclusion and exclusion criteria and performed the data extraction. Any discrepancies between the reviewers in either the decision of inclusion or exclusion of studies or in data extraction were resolved by discussion aimed at reaching consensus among all.

### Selection (inclusion/exclusion criteria)

Individual- or cluster-randomized controlled trials of routine (i.e. daily or weekly) zinc supplementation administered to children less than 5 years of age in developing countries.

• Studies with zinc supplementation carried out for 3 months of intervention in both intervention and comparison groups.

• Trials in which other nutrient co-interventions [e.g., vitamin A, riboflavin, iron-folic acid (IFA)] were administered to both control and zinc arms were included. Results were analyzed with and without studies in which zinc was given with IFA.

• Studies on small-for-gestational age or low birth weight infants were excluded from the review.

### Validity assessment

The quality assessment of each study was carried out on key variables with regard to study design, study limitations, intervention specifics, and outcome specific and graded according to the adapted GRADE technique [12,13]. Any study with a final grade of very low was excluded from the review. This review is shaped in large part by the needs of the LiST model. In this model, increases in coverage of an intervention results in a reduction of one or more cause-specific deaths or in reduction of a risk factor. Therefore the reviews and the grade process used were designed to develop estimates of the effect of an intervention in reducing either a risk factor or a death due to specific cause. For more details of the review methods, the adapted grade approach or the LiST model, see the method’s paper[12].

### Data abstraction

Data from all studies that met final inclusion and exclusion criteria were abstracted into a standardised form for each outcome of interest [12]. Data extracted from each eligible study included the following primary outcome variables: total number of deaths in both arms, deaths due to specific infection (diarrhea, pneumonia or malaria), the total number of episodes of illness (diarrhea, pneumonia and malaria) in each arm, the total amount of person-time accumulated in each arm (reported as person-days) and incidence rate ratio (IRR).

### Quantitative data synthesis

Meta-analyses were generated for all-cause mortality and cause specific mortality of diarrhea, pneumonia and malaria in children under 5 years of age. Pooled estimates for incidence of diarrhea, respiratory disease and malaria were also generated. Data from cluster randomised trials were pooled with that of individual randomised trial. In this case, cluster adjusted values were used irrespective of method used by the primary authors. Generic inverse variance method of meta-analyses was used for pooling the data. The assessment of statistical heterogeneity among trials was done by visual inspection i.e. the overlap of the confidence intervals among the studies, and by the Chi square (P-value) of heterogeneity in the meta-analyses. A low P value (less than 0.10) or a large chi-squared statistic relative to its degree of freedom was considered as providing evidence of heterogeneity. The I^2^ values were also looked into, and an I^2^ greater than 50% was taken to represent substantial heterogeneity. In situations of substantial heterogeneity being present, causes were explored, sub-group analyses performed and random effects model was used. Results of pooled estimates are described as relative risk (RR) with 95% CI. All meta-analyses were conducted using the software RevMan version 5 [14]. Data, where available, were taken for zinc versus placebo/no treatment (control) groups and for zinc plus other micronutrient versus other micronutrient, making zinc supplementation the only difference between the intervention and control groups.

We followed standardized guidelines of Child Health Epidemiology Reference Group (CHERG) to get estimates of effectiveness of preventive zinc supplementation in reducing diarrhea and pneumonia specific mortality [12]. Application of CHERG rules is based on three components 1) the volume and consistency of the evidence; 2) the size of the effect, or risk ratio and 3) the strength of the statistical evidence for an association between the intervention and outcome. The detailed description and application of these rules to collective morbidity and mortality outcomes is provided in the results and discussion section.

## Results

### Trial flow

**Figure 1:**
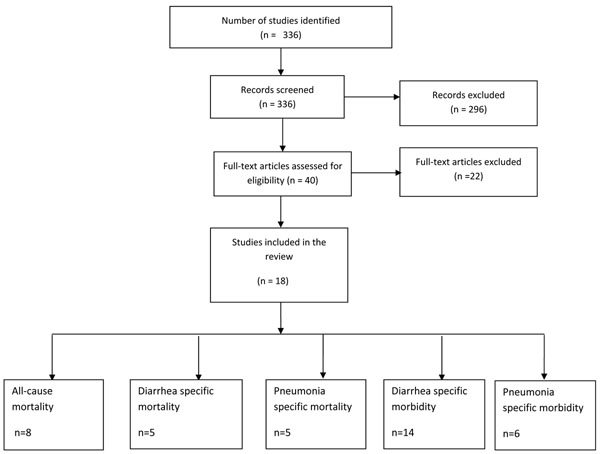
Flow diagram showing identification of studies.

The search on the electronic database and papers from hand searches yielded 336 titles out of which 18 studies were included in the present review [15-32] (Figure 1).

### Study characteristics

In the included papers, there were two cluster randomized controlled trials [30,31] and the remaining were individual randomized controlled trials [7,15-29,32,33]. All the trials assessing the effect of zinc supplementation were conducted in developing countries. There were 11 studies from Asia [16,18,20-23,25,26,30,31,33], 5 from Africa [17,19,24,27,28] and 3 from Latin America [15,29,32]. Participants in most of the studies were apparently healthy children. One trial included participants that had diarrhea in the past 24 hours [7,25], one included participants with persistent diarrhea [32], one trial enrolled stunted children [23] and in one study half of the children were stunted and half were non-stunted [24]. One study included participants older than 5 years of age [15]; however, disaggregated data on children < 5 years of age were available. One study on morbidity compared weekly supplementation versus daily supplementation with a placebo group, both the regimens were included in the analysis as two data sets (Gupta a , Gupta b) [16]. One study exclusively included HIV-positive children [28], while Luabeya et al. included HIV-positive, HIV-negative children born to HIV-positive mothers and HIV-negative children born to HIV-negative mothers [19].Additional File 1 gives the characteristics of included studies.

The dose of zinc supplementation ranged from 10 to 20 mg with a median of 10 mg. The duration of supplementation ranged from 4.6 to 18 months with a median of 6 months. Participants in all the studies were supplemented with zinc daily except in two studies where there was a weekly supplementation [16,21]. In three studies zinc was supplemented with multiple micronutrients [22,27,28]. Additional File 2 is the risk of bias table for all included trials according to the latest recommendations for the Cochrane Handbook.

### Quantitative data synthesis

#### All cause mortality

Mortality data was reported in ten papers comprising eight trials [17,19,21,26-28,30,31,34,35]. Of these, one was cluster RCT [30,34] and seven were individual RCTs [17,19,21,26,28,31,35]. One trial exclusively included infants < 12 months of age [21]; the other seven trials on mortality included children between 1 to 60 months of age [17,19,26,28,30,31,35]. Zinc was given with vitamin A in three trials [19,27,34], with multiple micronutrients in one [28] and alone in two trials [21,26]. But in all these studies, zinc was the only difference between the intervention and control groups. Three papers included IFA given in treatment and control arms [30,31,35].

Some studies were excluded from this analysis. The study on mortality by Sazawal et al [36] included only small for gestational age children, and the comparison was between zinc supplementation and supplementation with other micronutrients without any zinc, hence it was not included in the present analysis. The study by Lira et al. was excluded as supplementation was given for less than 3 months [37].

**Figure 2:**
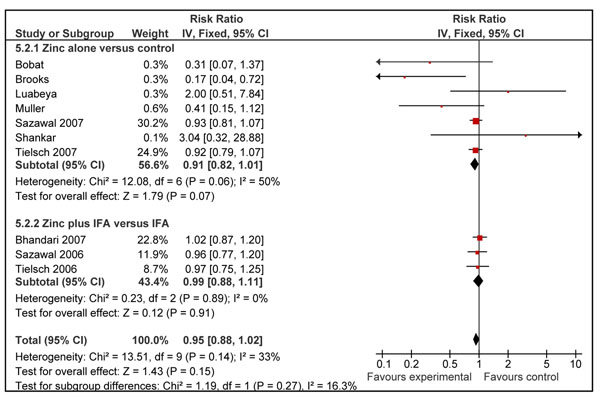
Effect of zinc supplementation on all-cause mortality in children less than five years of age

Zinc supplementation alone was associated with a statistically insignificant 9% (RR = 0.91; 95% CI: 0.82, 1.01; fixed effect model) reduction in all cause mortality in the intervention group as compared to controls using a fixed effect model (Figure 2). When studies in which zinc was given with iron and folic acid (IFA) [30,31,35] were included in the above analysis, the overall effect on mortality was reduced to 5% (RR = 0.95; 95% CI: 0.88, 1.02; fixed effect model), though also non-significant (Figure 2).

#### Cause specific mortality

Data on cause specific mortality due to diarrhea, pneumonia and malaria was provided among five trials consisting of six articles [21,27,28,30,31,34]. In all of these studies cause of death was usually ascertained through hospital records and verbal autopsies.

**Figure 3:**
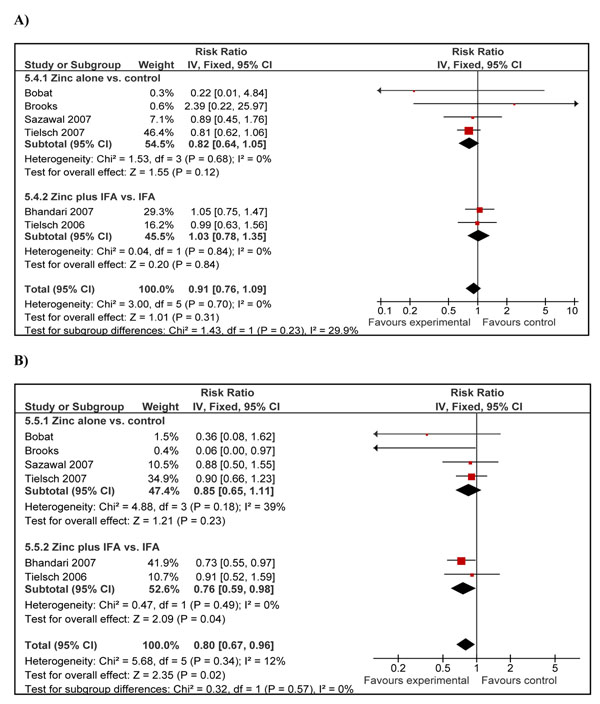
Forest plot for the effect of zinc supplementation on cause-specific mortality in children less than five years of age: A) due to diarrheal diseases B) due to pneumonia

Diarrhea specific mortality was presented in five trials consisting of six papers [21,27,28,30,31,34]. Analysis showed a non-significant 18% reduction in deaths due to diarrheal diseases (RR = 0.82; 95% CI: 0.64, 1.05; fixed effect model) among studies using zinc supplementation alone (Figure 3A). The effect decreased to 9% but remained insignificant (RR = 0.91; 95% CI: 0.76, 1.09) after inclusion of the two studies [30,31] with IFA supplementation as co-intervention in both the treatment and control arms. According to CHERG rules, we took the estimate of cause specific mortality reduction based on zinc supplementation only studies. Using the modified GRADE criteria, we rated the qualitative assessment for the above outcomes as low quality [12]. According to CHERG rules for evidence review, low quality evidence on cause specific mortality cannot be used as an effectiveness estimate on mortality reduction for an intervention and other outcomes should be reviewed [12].

Cause specific mortality data due to pneumonia were included in five trials presented in six papers [21,27,28,30,31,34]. Analysis of zinc supplementation only trials showed a 15% non-significant reduction in pneumonia specific mortality (RR = 0.85; 95%: CI 0.65, 1.11) in zinc recipients compared to controls. Further inclusion of studies with IFA [30,31] showed a 20% reduction in pneumonia specific mortality which did reach statistical significance (RR = 0.80; 95% CI: 0.67, 0.96) (Figure 3B). In one study [21] there were no deaths due to pneumonia in the zinc arm and 10 deaths in the control arm; the results remained consistent with reduction in mortality due to pneumonia after exclusion of the mentioned study. According to CHERG rules for evidence review, the level of evidence in support of zinc supplementation affecting pneumonia mortality in under 5 children was of low quality, as the overall results from trials of zinc supplementation alone on pneumonia mortality were statistically non-significant.

Malaria specific mortality data were provided in one large trial from Africa with zinc supplementation compared to placebo [27]. This was a large randomized controlled trial, with an overall high grade given to the individual study. There were 272 deaths in the zinc supplementation arm and 302 deaths in the control arm, and a 10% non-significant reduction in malaria specific mortality was observed (RR = 0.90; 95% CI: 0.77, 1.06). As the above data was provided from a single study the quality of *overall* evidence was rated as low.

#### Cause-specific morbidity impact estimation

Morbidity data on incidence of diarrhea were presented in 14 trials that met our eligibility criteria [15-25,28,29,34]. Generally, the age of the included participants ranged from newborn healthy children aged 5 years of age. In the study by Richard et al. children up to 15 years of age were included, however disaggregated data were available for children < 5 years of age [15]. In the study by Long et al. there were four groups: zinc, vitamin A plus zinc, vitamin A and placebo [29]. We were unable to compute risk ratio for zinc plus vitamin A versus vitamin A group and so used the risk ratio for zinc versus placebo only. Studies where zinc was given with IFA were not considered for any of the morbidity analyses.

**Figure 4:**
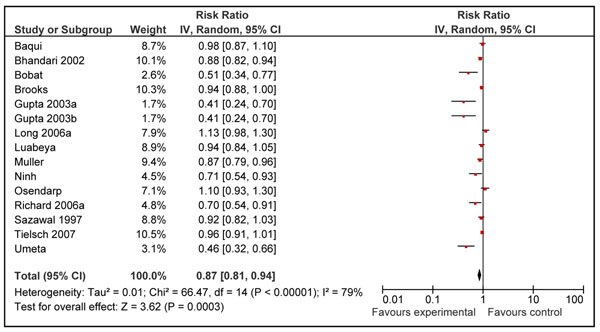
Forest plot for the effect of zinc supplementation on diarrhea morbidity (incidence)

The pooled analysis showed a significant reduction of 13% (RR = 0.87; 95% CI: 0.81, 0.94); random effects model) on the incidence of diarrheal episodes in zinc supplemented group as compared to control group (Figure 4). Hospitalization due to diarrheal episodes was reported by one study [19] with less than 50 events and, therefore, could not be included in the review as per CHERG rules [12].

**Figure 5:**
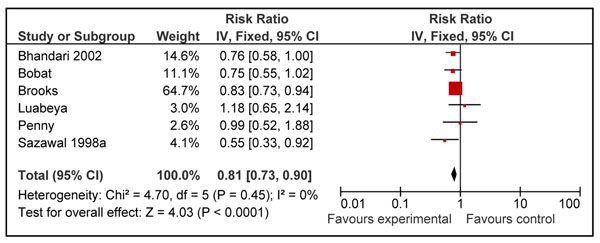
Forest plot for the effect of zinc supplementation on pneumonia morbidity (incidence)

The effect of zinc supplementation on pneumonia morbidity (definition rapid respiratory rate with or without other signs of pneumonia) was estimated from six RCTs [7,19,21,28,32,33]. Three RCTs [15,18,22] and data from one cluster RCTs [34] were not included in this morbidity analysis because the diagnostic signs used did not clearly identify pneumonia or depended upon self reporting by the patient. The analysis showed a 19% reduction in pneumonia morbidity in children who received zinc as compared to control (RR = 0.81; 95% CI: 0.73, 0.90) (Figure 5).

Four studies [9,15,17,26] evaluated the effect of zinc supplementation on incidence of uncomplicated malarial episodes with parasitemia. The pooled estimate of effect was an 8% (RR 0.92; 95% CI 0.82, 1.04) reduction in occurrence of malarial episodes in the zinc group as compared to control.

## Discussion

The benefits of preventive zinc supplementation are well established on morbidity related to infections, however; there has been no attempt to quantify the effect of preventive zinc supplementation on cause specific mortality in children which is a new aspect in our review compared to previous reviews on the subject. In their review of preventive zinc supplementation, Brown et al. [38] pooled data on children across all age groups (infants, preschoolers and older prepubertal children) and reported a 6% non-significant reduction in all-cause mortality. They also indicated that the mortality reduction with zinc supplementation versus placebo was only significant (18%) among children older than 12 months of age, with a tendency towards increased mortality in children less than 12 months. We, on the other hand, have evaluated the effect of zinc on all cause mortality, cause specific mortality and cause specific morbidity in children less than 5 years of age from developing countries receiving preventive zinc supplementation for a minimum period of three months. The studies of shorter duration were excluded as preventive zinc supplementation may require longer duration of supplementation to replete the body stores compared to that of therapeutic supplementation for diarrhea/pneumonia where two weekly doses are enough to fulfill the acute deficiency. Our results are similar to those reported by Brown et al., even though we restricted to the studies with children less than five years of age. Our 9% reduction in all-cause mortality was also non-significant. In our review, for the LiST model, estimates are presented from zinc only studies compared with placebo/no treatment, which may not be applicable to countries where there are on-going national supplementation programs of iron-folate, for example, India.

Table 1 gives grade quality of overall evidence. Zinc supplementation alone was associated with non-significant reductions in mortality due to diarrhea, pneumonia and malaria. Table 2 summarizes the application of CHERG rules to estimate effects for diarrhea and pneumonia specific mortality [12]. Zinc supplementation was associated with an 18% (RR = 0.82; 95% CI: 0.64, 1.05) reduction in deaths due to diarrheal diseases in under 5 children from developing countries. However, as the above values were statistically non-significant the evidence on cause specific mortality was labeled as low quality. In looking at severe morbidity estimates as surrogate measures, such as hospitalization due to diarrhea, this information was only available from one study with an overall event rate of less than 50 [12]. As per CHERG rules, we, therefore, considered the impact of zinc supplementation on diarrheal incidence for a point estimate of impact on diarrhea mortality as the evidence was of moderate quality[12]. With the above outcomes we took the most conservative effect between the available low quality evidence on diarrheal specific mortality and moderate quality evidence on mild morbidity. Using this sequential application of CHERG rules to the above outcomes, the observed 13% reduction (RR = 0.87; 95% CI: 0.81, 0.94) in diarrhea incidence was taken as an effect estimate for the protective effect of zinc supplementation on diarrheal disease mortality and recommended for inclusion in LiST tool [12].

**Table 1:** Quality assessment of overall evidence for effect of zinc supplementation (alone) in reducing morbidity and mortality in children > 5 years of age in developing countries

No of studies (ref)	Study Design	Limitations	Consistency	Generalizability to Population of Interest	Generalizability to intervention of interest	Relative Risk (95% CI)
***Outcome: All-cause mortality: Quality of evidence: Low***						

7	RCTs	Sequence generation and allocation concealment was unclear in few of the included studies	I^2^= 50%	Yes (all studies were conducted in developing countries)	The median dose of supplementation was 10 mg/day and median duration of supplementation was for 6 months.	0.91 (0.82-1.01)

***Outcome: Diarrhea specific mortality: Quality of evidence: Low***						

4	RCTs	Allocation concealment was unclear in two of the included studies	I^2^=0%	Yes (all studies were conducted in developing countries)	The median dose of supplementation was 10 mg/day and median duration of supplementation was for 6 months.	0.82 (0.64-1.05)

***Outcome: Diarrhea specific morbidity: Quality of evidence: Moderate***						

14	RCTs	Sequence generation and allocation concealment was unclear in few of the included studies	I^2^=79%	Yes (all studies were conducted in developing countries)	The median dose of supplementation was 10 mg/day and median duration of supplementation was for 6 months.	0.87 (0.81-0.94)

***Outcome: Pneumonia specific mortality: Quality of evidence: Low***						

4	RCTs	Allocation concealment was unclear in two of the included studies	I^2^= 39%	Yes (all studies were conducted in developing countries)	The median dose of supplementation was 10 mg/day and median duration of supplementation was for 6 months.	0.85 (0.65-1.11)

***Outcome: Pneumonia specific morbidity: Quality of evidence: Moderate***						

6	RCTs	Sequence generation and allocation concealment was unclear in few of the included studies	I^2^=0%	Yes (all studies were conducted in developing countries)	The median dose of supplementation was 10 mg/day and median duration of supplementation was for 6 months.	0.81 (0.73-0.90)

***Outcome: Malaria specific mortality: Quality of evidence: Low***						

1	RCT	None	NA	Study conducted in Zanzibar	Dose of supplementation was 10 mg/dl for children > 1 year and 5mg/day for children < 1 years.	0.90 (0.77-1.06)

***Outcome: Malaria specific morbidity: Quality of evidence: Low***						

4	RCTs	Allocation concealment was unclear in two of the included studies	I^2^=0%	Yes (all studies were conducted in developing countries)	The median dose of supplementation was 10 mg/day and median duration of supplementation was for 6 months.	0.92 (0.82-1.04)

**Table 2:** Application of standardized rules for choice of final outcome to estimate effect of zinc supplementation on mortality due to diarrhea, pneumonia and malaria in children less than 5 years of age

Outcome measure	Studies	Relative risk reduction	Application of standard rules
Cause specific mortality (diarrhea)	4	18% reduction; RR = 0.82 (0.64, 1.05)	(Low quality of evidence)
Incidence of diarrhea	14	13% reduction; RR = 0.87 (0.81, 0.94)	Rule 6 is applied (Moderate quality of evidence) (Effect Recommended for LiST)
Cause specific mortality (pneumonia) (*Zinc only studies*)	4	15% reduction; RR = 0.85 (0.65, 1.11)	Rule 6 applied (Low quality of evidence) (Effect Recommended for LiST)
Pneumonia morbidity	6	19% reduction; RR = 0.81 (0.73, 0.90)	Rule 6 applied (Moderate quality of evidence)

A similar effect of zinc was shown for pneumonia. Zinc only supplementation studies revealed a pooled effect size of 15% (RR = 0.85; 95% CI: 0.65, 1.11) reduction on pneumonia mortality but the overall quality of evidence was rated as low. We applied CHERG rules on studies with information on pneumonia morbidity outcomes where the overall evidence quality was rated as moderate. Applying CHERG rules we used the most conservative impact estimate from available outcomes i.e. 15% as our pooled surrogate effect size for mortality[12]. Deaths due to malaria were included only in one study which reported a 10% (RR = 0.90; 95% CI: 0.77, 1.06) reduction in malarial mortality (low quality of evidence). In the present review there was insufficient evidence of a protective effect of zinc supplementation on malarial mortality or morbidity. Therefore no estimates can be given for effect of zinc supplementation on reduction of deaths due to malaria for inclusion in the LiST tool.

Our review did not give any conclusive evidence about any possible positive or negative interaction between zinc and iron whereby iron decreases the absorption or bioavailability of zinc. With the inclusion of IFA studies for all-cause mortality, the results remained non-significant. For diarrhea-specific mortality, with inclusion of IFA studies, the impact estimate of reduction decreased from 18% to 9% but the results in both the situations were statistically non-significant. For pneumonia-specific mortality, however, a beneficial effect was seen as the impact of all the studies came out to be significant (RR = 0.80; 95% CI: 0.67, 0.96) compared to a 15% non-significant reduction with zinc alone studies (RR = 0.85; 95% CI: 0.65, 1.11).

To conclude, the application of CHERG rules to available evidence on diarrhea and pneumonia for providing effect estimates of protective effect for zinc supplementation in children for a minimum period of 3 months indicates 13% (RR = 0.87; 95% CI: 0.81, 0.94) reduction in mortality due to diarrheal diseases and 15% (RR = 0.85; 95% CI: 0.65, 1.11) reduction in pneumonia specific mortality. Our analysis supports inclusion of preventive zinc supplementation in public health programs to improve child health and survival and the suggested point estimates provide a suitable starting point for inclusion within the LiST tool and further program evaluation in effectiveness settings.

## Competing interests

We do not have any financial or non-financial competing interests for this review.

## Authors' contributions

Professor Zulfiqar A Bhutta developed the parameters for the review and secured requisite support. Drs Mohammad Yawar Yakoob, Evropi Theodoratou, Afshan Jabeen, Aamer Imdad and Thomas P Eisele, Joy Ferguson, Arnoupe Jhass and Igor Rudan participated in literature search, data extraction and writing of the manuscript under the overall supervision of Professor Bhutta. All authors contributed to the critical review and finalization of the manuscript.

## Supplementary Material

Additional File 1 Characteristics of included studies Click here for file

Additional File 2 Risk of bias table for the included studies according to the latest recommendations of the Cochrane Handbook Click here for file
